# miRge - A Multiplexed Method of Processing Small RNA-Seq Data to Determine MicroRNA Entropy

**DOI:** 10.1371/journal.pone.0143066

**Published:** 2015-11-16

**Authors:** Alexander S. Baras, Christopher J. Mitchell, Jason R. Myers, Simone Gupta, Lien-Chun Weng, John M. Ashton, Toby C. Cornish, Akhilesh Pandey, Marc K. Halushka

**Affiliations:** 1 Department of Pathology, Johns Hopkins University School of Medicine, Baltimore, Maryland, United States of America; 2 McKusick-Nathans Institute of Genetic Medicine, Johns Hopkins University School of Medicine, Baltimore, Maryland, United States of America; 3 Genomics Research Center, University of Rochester, Rochester, New York, United States of America; 4 Department of Biological Chemistry, Johns Hopkins University School of Medicine, Baltimore, Maryland, United States of America; University of Nevada School of Medicine, UNITED STATES

## Abstract

Small RNA RNA-seq for microRNAs (miRNAs) is a rapidly developing field where opportunities still exist to create better bioinformatics tools to process these large datasets and generate new, useful analyses. We built miRge to be a fast, smart small RNA-seq solution to process samples in a highly multiplexed fashion. miRge employs a Bayesian alignment approach, whereby reads are sequentially aligned against customized mature miRNA, hairpin miRNA, noncoding RNA and mRNA sequence libraries. miRNAs are summarized at the level of raw reads in addition to reads per million (RPM). Reads for all other RNA species (tRNA, rRNA, snoRNA, mRNA) are provided, which is useful for identifying potential contaminants and optimizing small RNA purification strategies. miRge was designed to optimally identify miRNA isomiRs and employs an entropy based statistical measurement to identify differential production of isomiRs. This allowed us to identify decreasing entropy in isomiRs as stem cells mature into retinal pigment epithelial cells. Conversely, we show that pancreatic tumor miRNAs have similar entropy to matched normal pancreatic tissues. In a head-to-head comparison with other miRNA analysis tools (miRExpress 2.0, sRNAbench, omiRAs, miRDeep2, Chimira, UEA small RNA Workbench), miRge was faster (4 to 32-fold) and was among the top-two methods in maximally aligning miRNAs reads per sample. Moreover, miRge has no inherent limits to its multiplexing. miRge was capable of simultaneously analyzing 100 small RNA-Seq samples in 52 minutes, providing an integrated analysis of miRNA expression across all samples. As miRge was designed for analysis of single as well as multiple samples, miRge is an ideal tool for high and low-throughput users. miRge is freely available at http://atlas.pathology.jhu.edu/baras/miRge.html.

## Introduction

MicroRNAs (miRNAs) are short (17–24 bp) RNA species that regulate translation across most species [1]. Identifying, characterizing and quantifying miRNAs has been an active area of research for a decade and has culminated in the creation of miRBase, a repository of known miRNAs [2]. The current version of miRBase (v21) contains 35,828 mature miRNA products across 223 species and is particularly rich in miRNA sequences from humans and model organisms such as mouse and rat. High-throughput profiling of miRNAs in biologic samples has historically been performed by qRT-PCR and hybridization arrays [3]. However, the popularity of RNA sequencing (RNA-seq) for miRNA profiling has risen as the cost of sequencing has decreased. RNA-seq is ideal as it allows the characterization of all known and unknown miRNAs, including isomiR forms, from a given RNA source. This advantage is tempered by the need for significantly more starting material than is necessary for qRT-PCR based approaches. A variety of RNA-seq computational tools exist, each with certain advantages and limitations, without consensus on an optimal method. This has created an opportunity for a new generation of fast and accurate tools to quantitate, annotate, and summarize the resulting data of each miRNA species from a sequencing run [3]. In particular, as more miRNA RNA-seq data is reported, there has become a greater appreciation of isomiRs and the need to identify them in RNA-seq datasets [4].

**Fig 1:**
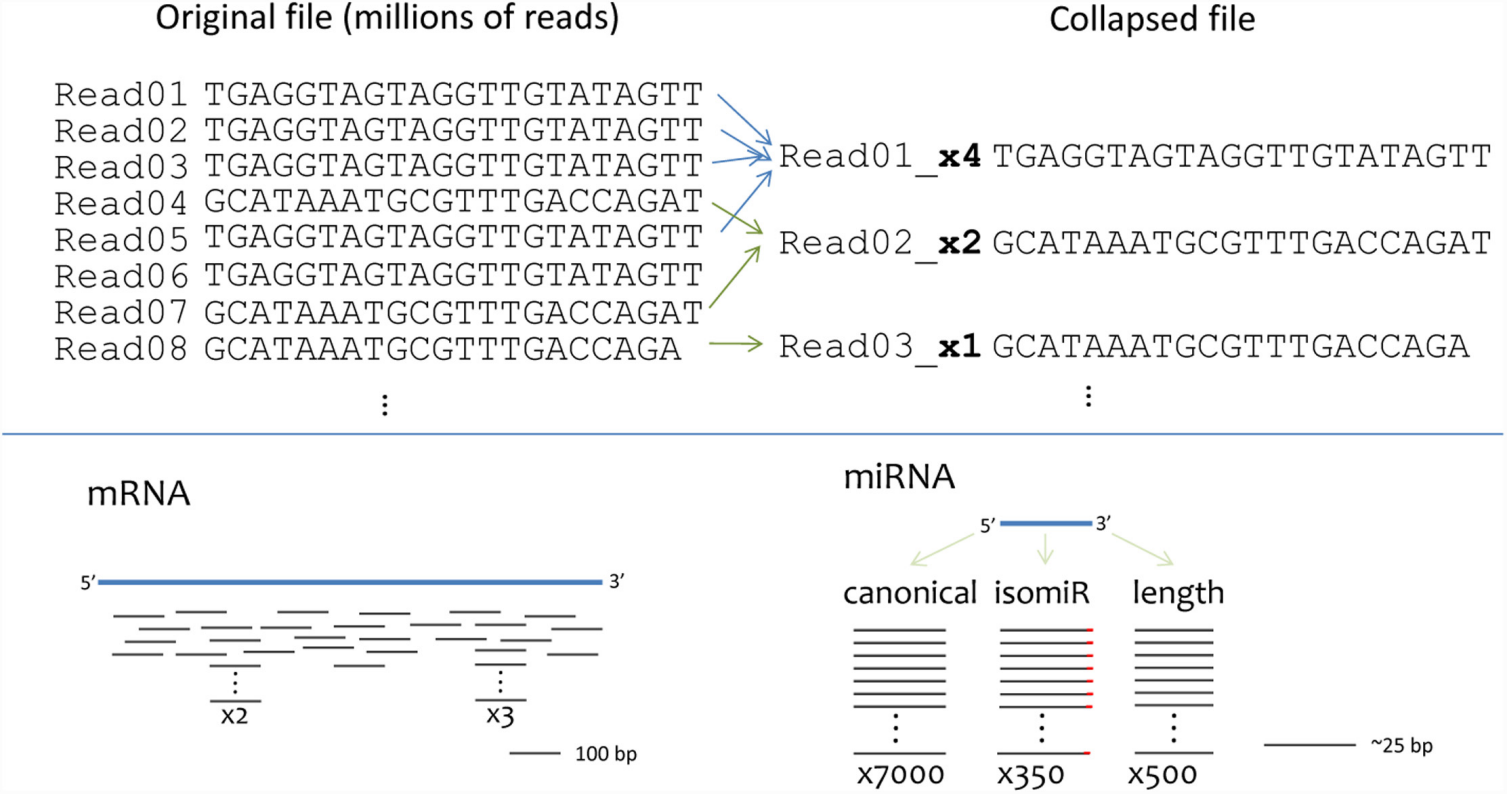
The benefits of collapsing reads in short RNA-seq data. Collapsing identical reads is advantageous for miRNAs because the species length (17-24bp) is less than the sequence length (50 bp). Collapsing is not advantageous for mRNAs or DNA.

Some features of miRNAs make their characterization from RNA-seq data easier than characterizing mRNA RNA-seq data. The major feature of miRNA RNA-seq that can be taken advantage of is the shorter read length of the miRNA (19-23bp) relative to the sequencing reads length (35–50 bp). This reduces quantitation to the enumeration of the unique nucleotide sequence elements present, which is in contrast to mRNA or genomic next generation sequencing (NGS) analytic approaches (Fig 1). A second feature of miRNA RNA-seq that we take advantage of is the relatively limited number of miRNAs described in any one species. In *Homo sapiens*, miRBase v21 lists 2,588 unique miRNAs. We and others have found most samples only contain up to ~350 reasonably expressed miRNAs [5–9]. This greatly simplifies the identification of RNA species relative to explaining the expression of >10,000 mRNAs. The analysis of mRNA must also consider gene length when normalizing to reads per kilobase of exon per million mapped reads (RPKM) or fragments per kilobase of exon per million mapped reads (FPKM) [10]. In contrast, miRNAs are essentially the same length, making a reads per million miRNA reads (RPM) value a simpler way to normalize the sequencing data.

MicroRNA RNA-seq data also has unique challenges, including the presence of isomiRs. IsomiRs are a collection of miRNA length and sequence variants due to imperfect editing by Dicer and RNA editing enzymes [11,12]. Imperfect Dicer editing can add additional hairpin nucleotides or reduce the length of the miRNA, most commonly on the 3’ end. Multiple nucleotides, predominately adenosines adenosine and uracil, can be added with preference to the 3’ end by RNA editing enzymes. These events result in hundreds of isomiRs for many miRNAs [12]. Because miRNAs are only 19–21 bp in length their sequences are likely to have higher alignment identity to random sequences, compared to mRNAs, found throughout the genome. This makes their proper assignment based on alignment to the entirety of the genome difficult and inaccurate with spurious alignments occurring in non-transcribed regions. This is particularly true for tools that align to the genome and have loose alignment parameters to allow isomiR assignments.

miRNAs also have a complex evolutionary background in which over 50 hairpin precursor miRNAs are found in two or more loci in the human genome. These loci produce identical mature miRNAs but often have variable nucleotides adjacent to the mature sequence. Additionally, many miRNAs are in families that result from duplications in which only a nucleotide or two are altered. For example, hsa-miR-107 and hsa-miR-103a-3p differ only at the 22nd nucleotide. This makes it challenging to disambiguate a sequence that is < 22 nucleotides in length and matches these two miRNAs equally.

miRNA entropy can be utilized to explain the diversity of isomiRs resultant from variable processing and imperfect editing. The spectrum of miRNA species that can result from the processing of a pre-miRNA can be interpreted in the information theoretic notion of entropy in which greater entropy would suggest more order to the system [13]. However, an accurate assessment of entropy is dependent upon reliable accounting of miRNA isomiR species.

Many groups have developed tools to characterize miRNAs by RNA-seq. These tools take short RNA-seq data from a variety of sources, output it in tabular or visual forms, and solve for the miRNAs contained therein. Numerous approaches utilizing a variety of database and alignment processes have been created [14–17]. These methods have not necessarily reconciled the unique nature of miRNAs to their alignment strategies. These samples are enriched for RNA species between 19 and 24 base pairs, and thus primarily represent miRNAs and their isoforms. Therefore, random genomic sequences which may be equivalent or better matches than isomiRs are overweighted in alignments to the genome and lead to underrepresentation of miRNAs. Additionally, the tools that do not align to the genome, but rather all RNAs, also do not give appropriate treatment to miRNA such as correcting for known miRNA SNPs. Moreover, all of these tools will deem any miRNA as valid, even if every aligned sequence is a non-canonical isomiR with one or more nucleotide changes. While complete RNA editing can occur, it has yet to be shown in a miRNA [18].

We aimed to improve upon existing tools with a highly parallelized, efficient, and rational analytic pipeline designed to accurately characterize short RNA-seq data with the goal of optimally capturing isomiRs to generate a comprehensive overview of miRNA expression. We herein describe miRge, a program designed to quantify miRNAs and other RNA species from small RNA-seq datasets obtained from modern sequencing systems. We provide head to head comparisons of miRge against other software tools to demonstrate its superiority in speed and miRNA assignment. We also demonstrate how miRge identified differential expression of isomiRs during stem cell maturation.

## Materials and Methods

### Bioinformatics sequence databases and software dependencies

Mature miRNA and miRNA hairpin libraries were obtained from mirBase.org (2). Human (*Homo sapiens*), mouse (*Mus musculus*), rat (*Rattus norvegicus*), fruitfly (*Drosophila melanogaster*), nematode (*Caenorhabditis elegans*) and zebrafish (*Danio rerio*) mRNA and other non-coding RNA libraries were obtained from Ensembl (www.ensembl.org/), unless otherwise denoted. Human tRNAs were obtained from the Genomic tRNA Database [19]. Human snoRNA was obtained from the snoRNABase (www-snorna.biotoul.fr/). miRge uses Cutadapt (https://github.com/marcelm/cutadapt) to trim linker sequences and perform sequence quality filtering [20]. Bowtie 1.1.1 (http://bowtie-bio.sourceforge.net/) is used to align reads to known sequence libraries [21].

### Search library preparation

miRge requires the use of four libraries: mature miRNA, hairpin miRNA, mRNA and other noncoding RNA (ncRNA). To optimize the alignment of miRNAs in miRge, several sequence libraries were modified. All mature miRNA sequences were brought up to a minimum of 25 nucleotides (useful for Bowtie alignment) by adding additional genome-accurate bases to the 5’ or 3’ ends. Generally two 5’ and six 3’ nucleotides were added to each miRNA. Additionally, 22 miRNAs that had identical sequence (or perfect complementarity) to another miRNA were removed from the human miRNA library (S1 File). Another 26 near-identical families were designated to be merged upon final reporting (S2 File). This was repeated in the mouse, rat, fruitfly, nematode and zebrafish with 15, 7, 4, 4 and 16 near-identical families that were merged upon final reporting (S3–S7 Files). Finally, all validated SNPs located in the mature regions of the human and mouse miRNA, as described in the microRNASNPdb, were added as additional searchable miRNA sequences to both human and mouse miRNA libraries [22].

Two records were removed from the human Ensembl cDNA database file (ENST00000244333 and ENST00000332503) as they contained the miRNAs hsa-miR-151a and hsa-miR-10a. From the mouse noncoding RNA fasta file obtained from Ensembl, all known miRNA records and an additional 14 records that contained miRNA sequences were removed (S8 File). All search libraries (mature miRNA, hairpin, coding RNA, and noncoding RNA) were made into Bowtie libraries using the bowtie-build command [21].

miRge was built to allow users to provide their own 4 respective reference annotations for sequence alignment to any species of interest. An additional automated script, miRge-build, will generate the appropriate bowtie libraries from user inputs for incorporation into miRge.

### miRge Workflow

**Fig 2:**
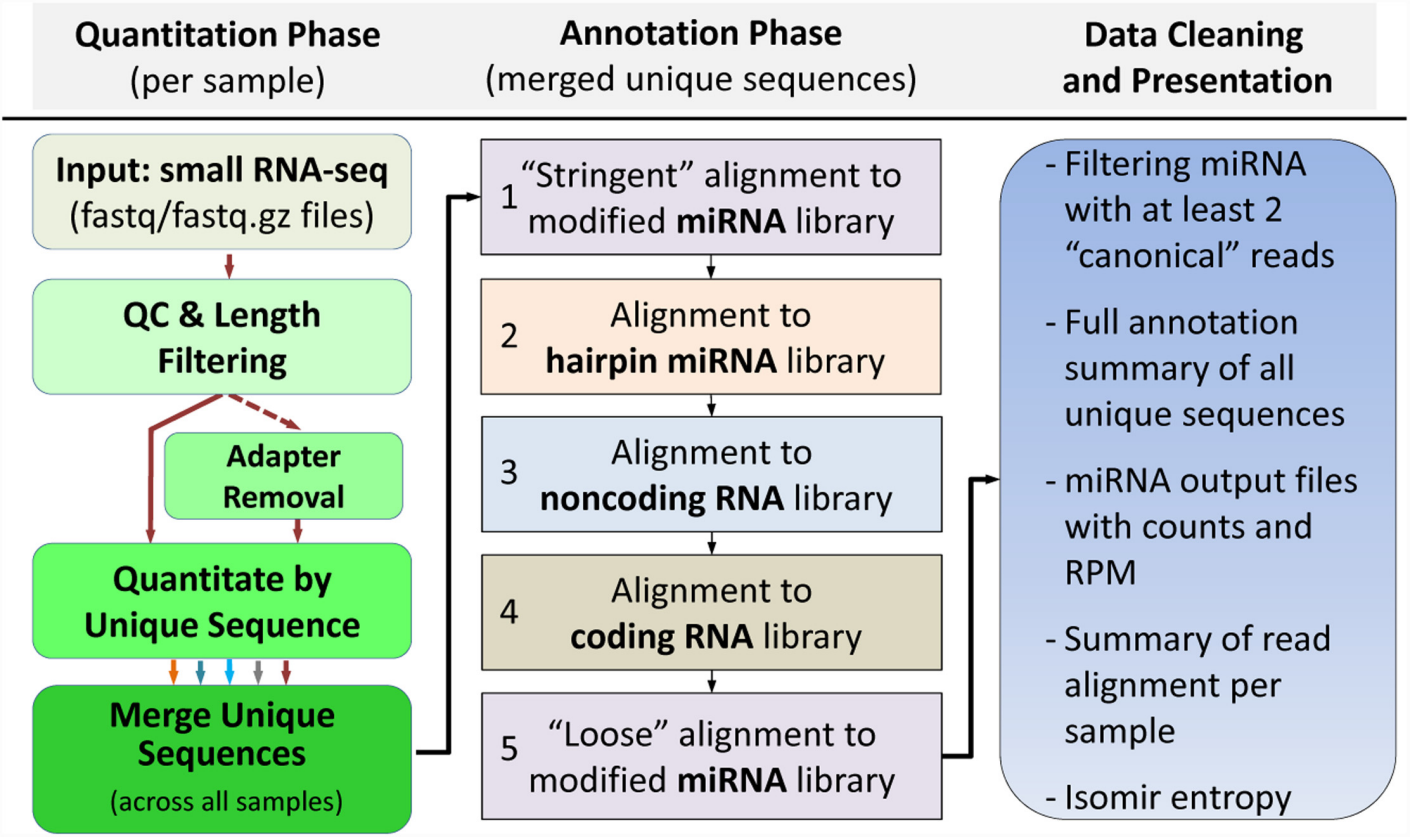
miRge: multi-sample quantization of unique sequences followed by a single sequential annotation method for miRNA-seq analysis. First, sequencing data undergoes a quality control and length filtering step. Sequences are trimmed of adaptors (optional) and unique sequences are quantitated per sample. The unique sequences identified across all samples examined then undergo 5 separate alignment steps against 4 libraries using Bowtie. Only reads > 25 bp are aligned to the hairpin miRNAs. The resulting data is organized and miRge outputs several files including a final miRNA oriented data table in both absolute counts and RPM.

miRge was designed to establish an efficient method of quantitation and a sequential method of annotation via sequence alignment. It is threaded, and thus can take advantage of available multi-CPU architecture during sample processing. miRge is comprised of three major steps: quantitation, sequential alignment, and data filtering along with organization (Fig 2). The quantitation begins with the raw fastq/fastq.gz files which are processed by Cutadapt to perform sequence quality filtering, sequence length filtering, and to remove sequencing linkers present [20]. The quantitation is then completed by tabulating the counts of all unique nucleotide sequences returned by Cutadapt identified across the set of samples. This set is then collapsed into a table of all unique reads and the number of each read by sample. Because miRNAs all have a length distribution of 16–25 bp, we begin the alignment pipeline with a “stringent” alignment to mature miRNA sequences for sequence elements ≤ 25 bps in length using Bowtie, allowing only identical matches of length 16–25 base pairs. Remaining unmatched nucleotide sequences are then aligned using Bowtie to the full hairpin miRNA for sequence elements > 25 base pairs in length. Remaining unmatched nucleotide sequences are then aligned to other noncoding RNA sequences including tRNA, snoRNA, and rRNA, allowing for a single nucleotide difference. Remaining unmatched nucleotide sequences are then aligned to coding RNA sequences (EST) allowing only for identical matches. In the last step to identify isomiRs, the remaining unmatched nucleotide sequences are again aligned to known miRNAs but in a less stringent manner to identify isomiRs, in which the first nucleotide and the last 3 nucleotides are ignored and up to 3 misaligned base pairs are allowed. This rational, step-wise alignment strategy for miRNA identification allows for a more accurate identification of isomiRs. This is because we adopt a triaged approach, where alternative alignments to various RNA species are excluded prior to a sequence being classified as an isomiR. Resulting alignments are then further filtered by requiring miRNA species to contain at least 2 reads from the “stringent” miRNA alignment step. This is based on the assumption that miRNAs do not exist in a purely “edited” form without canonical sequence or canonical sequence length variants present. Finally, miRge generates summary reports, which contain: 1) a file containing all aligned sequences with each row representing a unique nucleotide sequence; 2) a file containing all unaligned sequences with each row representing a unique nucleotide sequence (with the option to separate this file by sample identity); 3) a file containing reads summed per miRNA in which near identical miRNAs are also merged together (i.e. miR-103b/ miR-107, and miRNA SNPs); 4) a file as in 3 reported as RPM; 5) an optional file reporting miRNA entropy and % canonical reads per miRNA; 6) an optional file on the entropy of each isomir across samples and 7) an html file containing an annotation log of the unique sequences identified across the entirety of the sample set analyzed along with per sample information on total reads, sequence length histograms, and the composition of the sample with respect to miRNA, mRNA, ncRNA, genomic, and unaligned reads.

### Hardware

miRge, miRExpress (2.0), miRDeep2, and the UEA small RNA Workbench v3.2 were installed and run on a workstation with Dual Intel Xeon E5645 2.40GHz CPUs an ASUS Z8PE-D18 motherboard and 24GiB memory running Ubuntu 12.04 LTS. miRExpress was run with standard settings utilizing 5 processing cores. miRge was run utilizing 5 processing cores on the above system and was additionally run on a Dell Optiplex 9020 workstation with an Intel i7-4770 3.4GHz processor and 16 GiB of RAM running Ubuntu 14 LTS utilizing 6 of the processing cores. The UEA small RNAWorkbench v3.2 was run using 24 processing cores with the settings of 16 bp minimum length, 30 bp maximum length, minimum abundance 1, 2 mismatches allowed, aligning the genome and to miRBase v21, keeping only the best match and grouping mismatches. sRNAbench was run directly from the webserver to allow 2 mismatches, a seed length of 17, and a minimum read count of 2. omiRAs was run directly from the webserver with standard settings and a dummy second sample. Chimira was run directly from the webserver with adapter trimming and alignment analysis using standard settings. As the comparisons were designed to find known miRNAs, no novel miRNA discovery was performed in miRDeep2 and processing time was halted for sRNAbench before novel miRNA discovery.

### Evaluated data sets

For speed and alignment tests, we evaluated 103 short RNA-seq Illumina datasets obtained from the Sequence Read Archive (SRA). These were human adipose tissue (SRR772341-SRR772349; SRR772351-SRR772440; SRR77563), human beta cells (SRR873410), the miRQC study sample A (SRR950876) and a mouse liver sample (SRR947057) [9,23–25]. For miRNA entropy tests, we evaluated 16 files from SRA. These were normal pancreas and pancreatic cancer (ERR852089—ERR852099), and embryonic stem cells (ESC) maturing to retinal pigment epithelium (RPE) (SRR493011-SRR493015) [26,27].

### Statistical testing and correlation between datasets

We used the miRQC study sample A (SRR950876) as a benchmark to compare the 7 methods for miRNA identification. These data were compared to the data reported in the miRQC paper for the Illumina reads [23]. After identifying known miRNAs using each method, we performed Pearson correlations based on 333 shared miRNAs each with a minimum read count of 10 across all samples using R (version 3.1.1).

The entropy of a given miRNA was calculated as −$\sum \begin{array}{l} n\\ i\;=\;1 \end{array}\;p_{i}log_{2}\left(p_{i}\right)$ where *n* represents the number of unique miRNA species with read counts ≥2 from a given miRNA “family” and *p*_*i*_ represents the proportion of the reads mapping to a given miRNA “family” that are accounted for by a unique miRNA species with read counts ≥2. Different miRNA “families” can have a different total number of unique miRNA species (*n*). Therefore, the entropy as calculated above was normalized by the maximal possible entropy *log*_*2*_ *(n)* resulting in a normalized entropy scaled between 0 and 1. The assessment of normalized entropy was limited to miRNA “families” for which the most abundant unique species had a RPM of > 20 in order to allow for enough data points to support this calculation. Normalized entropy values across different samples were compared with Spearman correlations and Kolmogorov-Smirnov tests. The probability density function for normalized entropy within a sample was generated by kernel density estimation using a normal kernel with bandwidth 0.15. All statistical tests were performed using MATLAB 8.4 R2014b.

## Results

### miRge run time and results

We performed tests on miRge to profile its performance on small and large datasets. On a 2.37 million read (SRR772563) file of human adipose tissue small RNAs, miRge completed its analysis in 25 seconds (Table 1). This resulted in the discovery of 2.04 million miRNA reads representing 86% of all reads in the original sample size. Only 40,876 reads (2%) remained unaligned at the end of the analysis run, with other reads assigned exclusively to hairpins (1,373), mRNAs (5,302), and other RNAs (rRNAs, snoRNAs, tRNA pieces) (158,601).

**Table 1:** Profiling and miRNA assignment across 5 methods in 3 separate samples.

	Human Adipose Tissue (SRR772563)			
Method	Processing time	miRNA Reads	miRNAs	miRNAs >10 RPM
miRge	**26 sec**	2,041,334	479	245
miRExpress 2.0	3.5 min	1,503,704	593	240
omiRAs	14 min	1,672,612	458	238
miRDeep2	13.5 min	1,969,122	432	189
sRNAbench	7 min	1,916,307	969	278
Chimira	2 min	**2,044,664**	804	268
UEA small RNA Workbench	3.6 min	1,583,013	578	225
	Human Beta Cell (SRR873410)			
Method	Processing time	miRNA Reads	miRNAs	miRNAs >10 RPM
miRge	**5.2 min**	26,169,405	884	306
miRExpress 2.0	62 min	16,386,290	878	260
omiRAs	55 min	25,823,397	804	288
miRDeep2	39.5 min	19,949,196	489	196
sRNAbench	21 min	23,755,866	598	276
Chimira	24.4 min	**26,238,680**	1,499	323
UEA small RNA Workbench^a^				
	Mouse Heart (SRR402445)			
Method	Processing time	miRNA Reads	miRNAs	miRNAs >10 RPM
miRge	**2.7 min**	8,783,714	519	274
miRExpress 2.0	22.7 min	6,939,148	742	247
omiRAs	16 min	8,298,256	525	254
miRDeep2	13 min	6,336,341	529	216
sRNAbench	13 min	7,696,386	927	294
Chimira	10.2 min	**8,839,153**	893	265
UEA small RNA Workbench	9 min	4,497,946	583	226

Starting read counts: SRR772563 = 2,373,604 reads; SRR873410 = 33,233,648 reads; SRR9402445 = 15,981,680 reads. Each method was run with the number of processing cores reported: miRge—5 cores; miRExpress 2.0–5 cores; omiRAs—5 cores; miRDeep2–1 core; sRNAbench—unknown; Chimira—unknown; UEA small RNA Workbench—24 cores. Bold indicates fastest time and most miRNA reads.

^a^ unable to complete due to memory limitations.

We then performed miRge on a larger human beta cell sample (SRR873410) containing 33.23 million reads. This sample was processed in 5 minutes and we obtained 26.17 million miRNA reads (Table 1).

To evaluate miRge on a non-human source, we analyzed a mouse heart dataset (SRR402445) containing 15.98 million reads. This was processed in 2.6 minutes obtaining 8.78 million miRNA reads. Nearly 1.8 million reads were removed during the prealignment steps of linker removal, QC and collapsing reads. Combined rRNA, snoRNA and tRNAs accounted for 2,211,257 reads. mRNAs (98,602) and hairpin reads (4,482) were also captured. There were 1.2 million unaligned reads, of which > 500,000 reads were of a single 42 bp sequence that failed to align to the mouse genome (blastn search to RefSeq Genomic DNA).

Finally, to demonstrate the extreme multiplexing ability of miRge, we evaluated multiple RNA-seq files at once. We obtained 100 human adipose tissue small RNA-seq fastq files (SRR772341-SRR772349; SRR772351-SRR772440; SRR77563) with a combined 199,827,852 reads from SRA. These were processed through miRge in one batch, taking 52 minutes to complete. Filtering removed 27.5 million reads. Of the remaining ~172 million reads, we identified 151,196,677 miRNA reads (88% of all reads) from the QC and length filtered run.

### Comparisons with other methods

We compared miRge output to other well-described methods of miRNA analysis (Table 1). Because most miRNA analysis tools do not allow for multiple files at upload, we ran all samples independently. For the 2.4 million read human adipose tissue sample, miRExpress completed the run in 90 seconds, Chimira in 2 minutes and the UEA small RNA Workbench in 3.6 minutes. sRNABench, the improved version of the popular miRAnalyzer, completed the run in 7 minutes followed by miRDeep2 and omiRAs logging in at 13.5 and 14 minutes respectively. miRge identified more miRNAs in the sample than any of the other method except Chimira (Table 1). miRExpress (which does not identify RNA-edited isomiRs) identified almost 500,000 fewer reads. The number of miRNAs with a read count of 10+ RPM varied between methods (189–278, median 240).

In the 33.2 million read beta cell data set, miRge performed significantly faster than the other methods and again identified the second most miRNA reads. While miRge processed the reads in 4.5 minutes, the other methods ranged from 21–62 minutes (Table 1). miRge was also the fastest method (2.7 minutes) for a 16 million read mouse heart data set (SRR402445) and again found more miRNAs than every other method except Chimira. miRge was consistently 4 to 28-fold faster than all other methods. The 100 adipose tissue sample could not be compared to the other tools as none of them are capable of that level of multiplexing.

### Correlation between miRNA discovery methods

**Fig 3:**
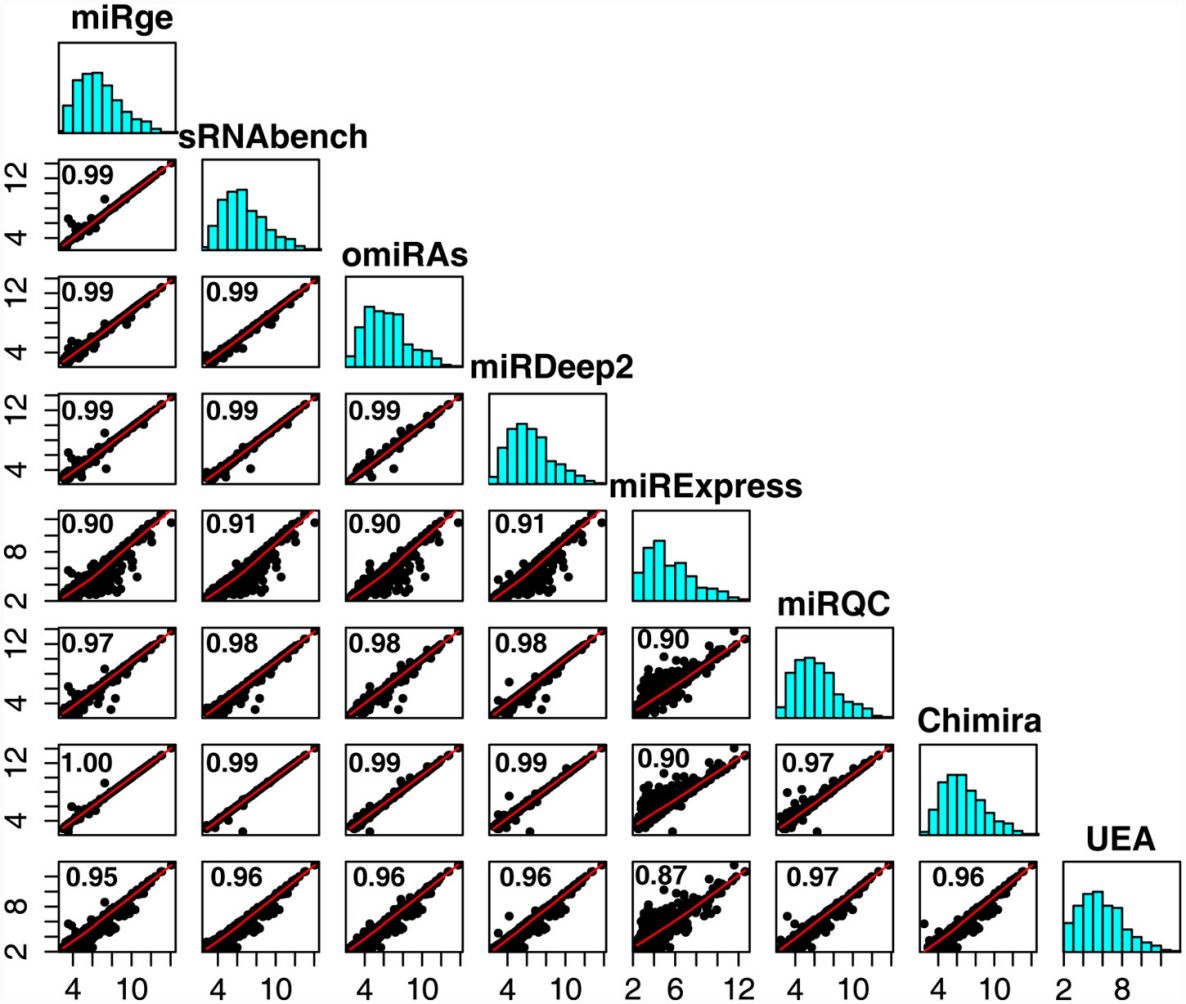
Comparisons across 8 methods of miRNA identification. The miRQC sample A RNA-seq Illumina data set was analyzed by 7 methods and compared to the original data. For each method, a histogram is given of log_2_ normalized miRNA read counts for 333 shared miRNAs. Pearson correlation was performed for each comparison and a scatter plot with loess curve is presented.

To determine if the superior speed and miRNA discovery of miRge resulted in an unusual selection of miRNAs discovered, we evaluated identifications of known miRNAs from the miRQC study sample A (SRR950876) using each of the 7 miRNA alignment tools. We compared these miRNA read counts to the Illumina method results provided in the miRQC manuscript [23]. By Pearson correlation we observed strong correlations between all of the methods (0.87–1.00) with only miRExpress and the UEA small RNA Workbench being outliers (Fig 3). These data indicate that miRge is consistent in reporting known miRNAs despite finding more miRNA reads than most other methods.

### Comparing miRge alignments to unique libraries versus the genome

Because we have proposed that aligning to individual RNA libraries is a better approach than aligning to the genome, we investigated how these alternative methods would affect isomiR discovery. For this experiment, we altered miRge to align directly to the genome (hg19) using our sequential “stringent” and “loose” searches. We performed miRge on the 33 million read human beta cell sample (SRR873410). In comparison to aligning to libraries, when aligning to the genome, we aligned more reads (28.7 vs. 25.9 million reads); however, the number of reads aligned to miRNAs was much lower (5.23 vs 7.82 million reads). When considering the number of isomiRs identified, for miRNAs with at least 100 reads we observed the median of isomiRs to be 110 in the standard miRge run but only 54 in the miRge run aligned to the genome. Thus, there was a 2 fold increase in isomiRs using the standard miRge approach.

### Characterization of the spectrum of miRNA entropy with respect to the biological degree of differentiation

We evaluated the extent to which the normalized entropy measure, as defined in our methods, was effected by the inclusion of unique miRNA species supported by only 1 read, i.e. singletons. At this low level of abundance we cannot distinguish whether these reads represent editing events or sequencing errors. Furthermore, we found that the inclusion of singletons significantly inflated the normalized entropy distribution towards higher disorder, as might have been expected (S9 Fig). Based on these findings, we limited the calculation of normalized entropy to include unique miRNA species with read counts of at least 2.

**Fig 4:**
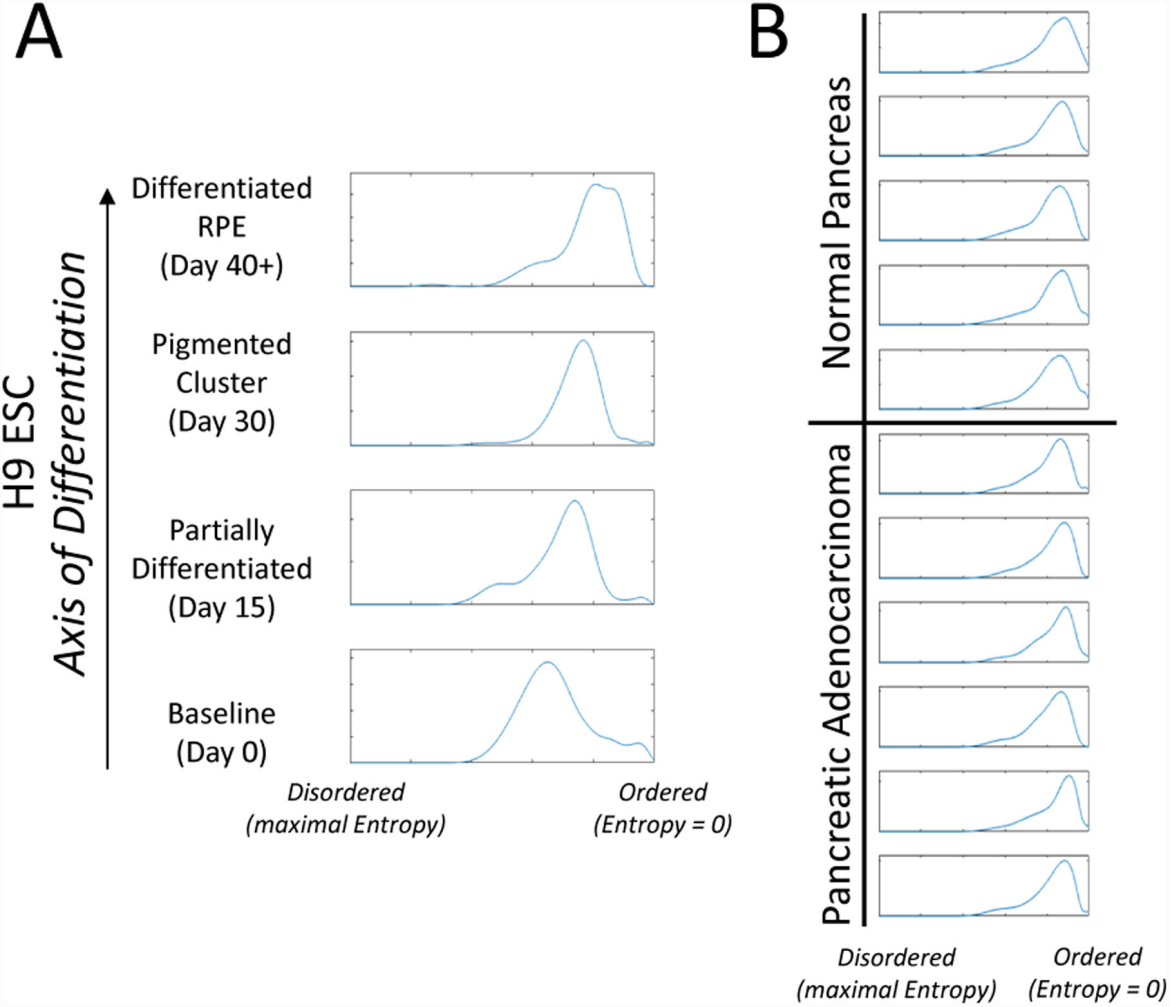
The spectrum of miRNA entropy. Kernel density estimates of the distribution of normalized miRNA entropy in two sample sets. **A)** As embryonic stem cells (ESCs) differentiate towards retinal pigment epithelial cells (RPE) the distribution of miRNA entropy is shifted towards more order (Spearman correlation coefficient 0.14, p>0.001). **B)** No significant difference in the distribution of miRNA entropy with respect to normal pancreas vs pancreatic adenocarcinoma is observed (Kolmogorov-Smirnov test p > 0.05).

We then postulated that normalized miRNA entropy may vary from more primitive states to more mature states. To test this, we compared the entropy of ESCs that were matured into RPEs over 30 days and compared them to RPE cells in culture for >3 months [27]. We noted a consistent decrease in entropy (i.e. increase in order) over the maturation of the samples, Spearman correlation coefficient 0.14, p<0.001) (Fig 4A). Given that it has been postulated that some malignancies may represent a “de-differentiated” state, we next compared six pancreatic adenocarcinoma samples to 5 normal pancreas samples [26]. Consistent with the data from the above cell line studies, the fully differentiated benign adult pancreatic tissue samples exhibited a high degree of “order.” However, in contrast to the maturation series in Fig 4A, we did not observe a significant difference when comparing the miRNA entropy spectrums across normal vs cancer tissues, Kolmogorov-Smirnov p>0.05 (Fig 4B), suggesting that this phenomenon is not effected by malignant transformation in these tissues

## Discussion

### The rationale of miRge

In creating miRge, we rationalized the ideal workflow for small RNA-seq analysis, then constructed the workflow from a new coding framework and a few established tools. Our workflow begins with the understanding that small RNA-seq samples that use the Illumina TruSeq Small RNA kit are size selected to capture only ~15–35 base RNA species. Thus, this sample should not generally contain genomic DNA or longer RNAs (mRNAs, lncRNAs, etc.) and there is a strong bias in the RNA sample for miRNAs. We demonstrated that when extensive miRNA libraries are known for a species, it is a better strategy to align to the library rather than the entire genome to maximize isomiR discovery. Aligning small RNA-seq sequences to the entire genome, where it may align in multiple coding and non-coding areas with equal probability, resulted in fewer proper miRNA assignments, likely due to isomiRs aligning to locations in the genome with a higher identity than to the mature miRNA locus.

The first decision in our workflow was to incorporate a step that collapses identical reads as has been done in miRDeep2, miRExpress, sRNAbench and other programs [14–17] (Table 2). Due to the shorter length of a miRNA (<26bp) relative to a read length (35-50bp), most short RNA-seq runs, composed predominately of miRNAs, will collapse to <10% of the original size. Where we have taken a different approach is first tabulating the counts for the unique sequences identified across a set of samples examined and only then annotating the set of unique sequences identified with respect to miRNA, mRNA, genomic, or other nucleotide sequences. To the best of our knowledge, this algorithmic approach is considerably more computationally efficient for RNA-seq data analyses than any prior software tools and scales well for larger data sets and multiplexed data sets. It is expected that the majority of sequence elements will be found to some extent across most samples of a given dataset. Therefore, it is highly redundant and inefficient to perform alignments on the unique sequence elements identified from each sample independently. Additionally, large sequencing facilities could consider storing the annotation results of all unique sequence elements encountered to further optimize computational efficiency.

**Table 2:** A comparison of common miRNA alignment methods.

			Method				
	miRge	sRNAbench	omiRAs	miRDeep2	miRExpress	Chimira	UEA small RNA Workbench
Map to	Modified libraries	Genome or libraries	Genome	Genome or libraries	Hairpin	Hairpin	Genome and/or mature
Input	Fastq, Fastq.gz	Fastq, Fastq.gz, sra^a^, read count^a^, Fasta^a^	Fastq, Fastq.gz	Fastq	Fastq	Fastq.gz, Fasta.gz	Fastq
Process multiple files	Yes	No	Yes (≤2GB)	No	No	Yes (≤2GB each)	Yes
Identify novel miRNAs	No	Yes	Yes	Yes	No	No	Yes
Identify other RNA species	Yes	Yes	Yes	Yes	No	No	No
Allows RNA edited IsomiRs	Yes	Yes	Yes	Yes	No	Yes	Yes
Incorporates miRNA SNPs	Yes	No	No	No	No	No	No
Visual outputs	Yes	Yes	Yes	Yes	Yes	Yes	No
Format	Stand-alone	Web based / Stand-alone	Web based	Stand-alone	Stand-alone	Web based	Stand-alone
Alignment Tool	Bowtie	Bowtie	Bowtie	Bowtie	Smith-Waterman algorithm^b^	BLASTn	PatMaN

^a^ Executable version only.

^b^ Smith-Waterman algorithm implemented following Single Instruction Multiple Data (SIMD) instructions

The annotation pipeline is designed to be a rational approach to the characterization of the considerable diversity of RNA-seq reads including miRNA/isomiRs, mRNA, noncoding RNA, and genomic contaminants. Utilizing a “stringent” first pass through all RNA species and a “loose” second pass through all RNA species allowed us to optimally identify isomiRs, as demonstrated by miRge's higher alignment counts than most other miRNA alignment tools. Additionally, we adjusted the miRBase miRNA reference file, adding two 5’ bases and up to six 3’ bases from the hairpin to each mature miRNA. This was to capture the entirety of length variants in miRNAs in the first step and is akin to other programs such as Chimira that align directly to the hairpin sequence. Known, validated SNPs were also included, as we discovered that a central SNP in the potentially highly expressed hsa-miR-28-3p significantly impacted its read counts in the presence of heterozygosity using other aligners. We reasoned SNP variants could impact on read counts for all miRNAs in miRge, due to the stringent alignment parameters.

Another unique feature of miRge is that it requires that all reported miRNAs have at least two alignments that match the canonical or length adjusted sequence (i.e. no RNA-editing or sequencing errors). Thus, we use the first “stringent” alignment to the miRNA library to obtain a list of all allowable miRNAs in a given sample and then expand the counts of each. Other miRNA bioinformatics tools report miRNAs that do not have any canonical reads, which frequently skew miRNA read results. The combined effect of our approach is to maximize the capture of true miRNAs and minimize false assignments due to sequencing errors.

miRge is most similar to the new miRNA tool Chimira. Chimira, based on the earlier Kraken tool, is web-based, has a very simple to use graphical user interface, internal analysis tools, and attractive graphics [28,29]. The two methods were comparable in miRNA calling having a pairwise value of 1.00 (Fig 3). Chimira identified more miRNAs due to two methodological differences. The first is that miRge reports any miRNAs >25bp and having up to 1 mismatch as a separate miRNA hairpin read. These are included in mature miRNAs reads in Chimira. Secondly, the “stringent” first step of miRge avoids any reads that do not have a canonical sequence. Chimira, and all other tools, do not have this step which resulted in it identifying some non-miRNAs as miRNAs. Another useful tool is sRNAbench, which is in the sRNAtoolbox [30]. It is also web-based, provides comprehensive analysis of the data, and performs new miRNA discovery. While miRge is the fastest tool, with unique features including the ability to determine miRNA entropy, there are other competing tools that also have appealing features.

We specifically avoided using the Rfam database for other RNA alignments as is used in miRDeep2 [31]. Rfam is a collection of RNA families represented by multiple sequence alignments, consensus secondary structures and covariance models. Although Rfam is a useful database for a variety of purposes, it unfortunately has a known problem with tRNAs, in which their database is both incomplete and has <40% accuracy to validated human tRNAs. This causes any alignment tool that uses Rfam, instead of a specific tRNA library, to significantly underrepresent this common RNA species in small RNA-seq data and potentially misassign other reads.

### miRNA entropy

miRge is the first alignment tool to actively determine miRNA entropy for the user. We used that function to demonstrate that entropy moves toward order as cells go from a more primitive state (ESC) to a mature cell type (RPE). This provides a hint that a cell’s RNA-editing may change over time and may reflect alterations in Dicer activity or other editing proteins. We also noted no significant difference in entropy between pancreatic cancer and normal samples, suggesting that despite much evidence of a more embryonic state for cancer cells, a change to greater entropy did not exist for isomiRs [32].

### Reporting miRNA RNA-seq data

One continuing question in RNA-seq is how to report on discovered miRNAs. It is generally thought that the presence of a single miRNA read out of 1+ million reads is likely of no functional significance. Mullokandov et al. suggested that a miRNA should be at a minimum of 100 RPM for functionality and that 80% of miRNAs over 1000 RPM were functional [33]. While not ideal, we support normalizing the data in RPM. We further believe that a RPM threshold of 10 RPM for reporting will identify all functional miRNAs while removing many inconsequential reads. To consistently detect miRNAs with a threshold of 10 RPM, the depth of a sequencing run need not be more than 3 million reads. This is a significant advantage when multiplexing samples together for cost effectiveness [34]. Recently, the miRQC consortium showed how miRNA discovery continues to increase as RNA-seq depth increases into the tens of millions of reads [23]. However, this report clearly includes singleton reads, which would increase, but are likely biologically irrelevant and, in the setting of tissue, may be a blood-based miRNA “passing through.”[7].

### miRge limitations

There are certain limitations to miRge. The current version of miRge does not attempt to perform any novel miRNA discovery. It should be noted, however, that the residual reads file can be used in other programs (ex. RNAfold) for that purpose [35]. The miRge algorithm and the sequence libraries provided were developed for human, mouse, rat, nematode, fruitfly and zebrafish datasets, but miRge can be used in any species in which the four sequence libraries utilized in the miRge workflow can be constructed. There is no limitation to individual users developing their own optimal datasets for a species of interest, as long as the miRBase miRNA coverage is adequate for that species. Finally, we, like others, have not yet solved the problem of assigning reads that can map equally to two miRNAs. We currently combine near identical miRNA species in our final reporting, which is also customizable via a simple comma separated text file. It will require *in vitro* experimental methods to determine the proper ratios of the miRNA sequences based on known differences between miRNAs and their independent isomiR ratios.

## Conclusion

We have created miRge, a new small RNA RNA-seq alignment program to rapidly and accurately determine the miRNA content of RNA-seq data and provide novel miRNA entropy measures.

## Supporting Information

S1 Fig miRNA entropy distribution can be biased by singleton reads. When all reads are included, a histogram of entropy value across all miRNAs in heavily skewed towards an entropy of 1. The removal of singletons reads markedly alters the overall histogram of entropy. Further changes in entropy measures by removing reads with 2 or 3 counts are negligible. These data are independent of the total number of miRNA reads in a sample between 1 million and 26 million (not shown).
(PDF)

S1 File Human miRNAs removed from the modified miRNA alignment library. These miRNAs were either inverted versions of known miRNAs or identical sequences of known miRNAs.
(XLSX)

S2 File 26 human near-identical miRNA families merged in final reporting. (XLSX)

S3 File 15 mouse near-identical miRNA families merged in final reporting. (XLSX)

S4 File 7 rat near-identical miRNA families merged in final reporting. (XLSX)

S5 File 16 zebrafish near-identical miRNA merged in final reporting. (XLSX)

S6 File 2 fruitfly near-identical miRNA merged in final reporting. (XLSX)

S7 File 2 nematode near-identical miRNA merged in final reporting. (XLSX)

S8 File Mouse mRNA records removed from the mRNA library for containing a miRNA. These ENSMUST files each contained miRNAs and were removed to avoid misalignments in the miRge workflow.
(XLSX)
