# Supplementary material for: miRge - A Multiplexed Method of Processing Small RNA-Seq Data to Determine MicroRNA Entropy
Source: PLoS One. 2015 Nov 16;10(11):e0143066. doi: 10.1371/journal.pone.0143066 (PMC4646525; doi:10.1371/journal.pone.0143066)
Supplement: S1 Fig — When all reads are included, a histogram of entropy value across all miRNAs in heavily skewed towards an entropy of 1. The removal of singletons reads markedly alters the overall histogram of entropy. Further changes in entropy measures by removing reads with 2 or 3 counts are negligible. These data are independent of the total number of miRNA reads in a sample between 1 million and 26 million (not shown). (PDF) [file pone.0143066.s001.pdf]

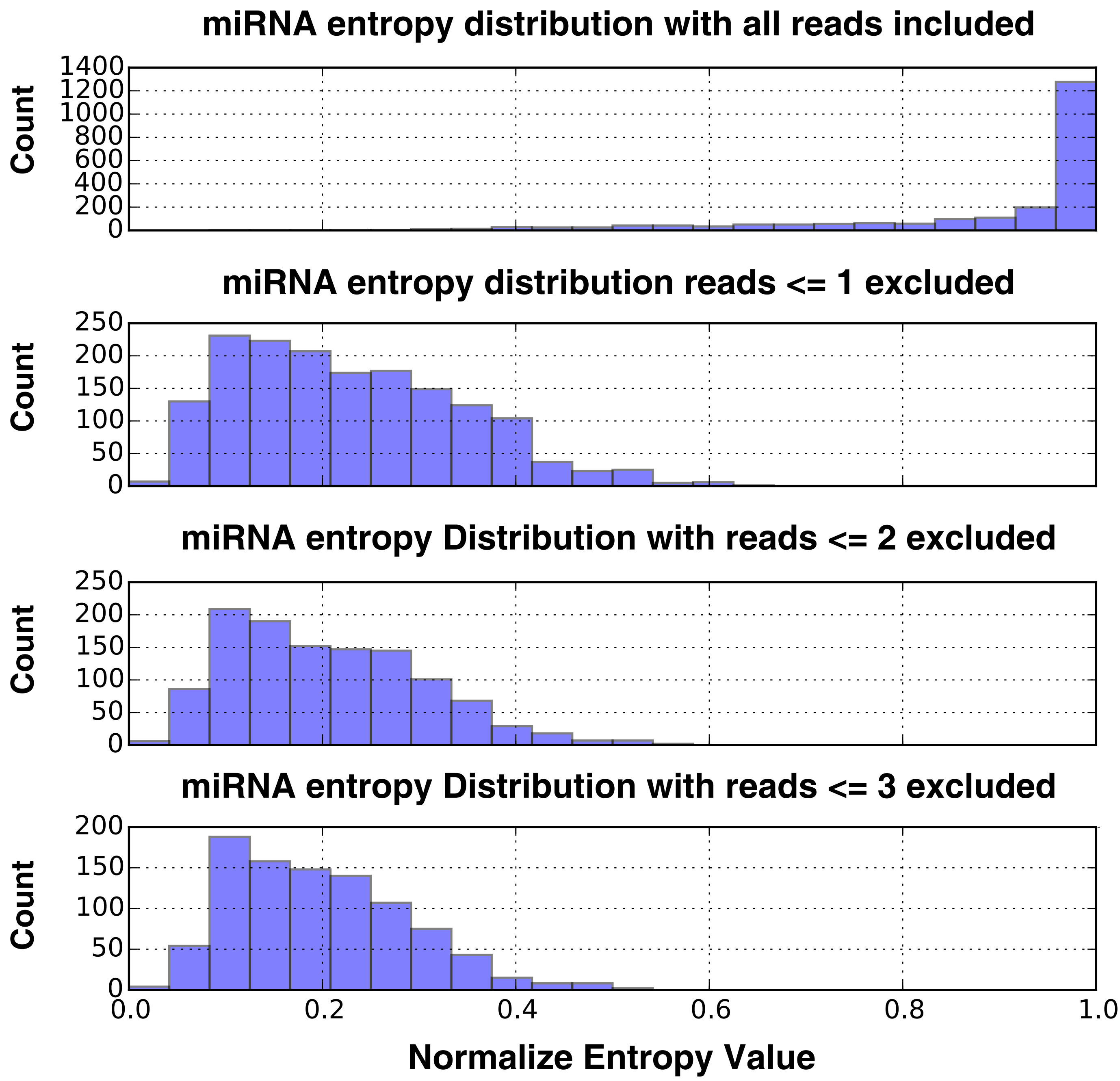
miRNA entropy distribution with all reads included
miRNA entropy distribution reads <= 1 excluded
miRNA entropy Distribution with reads <= 2 excluded
miRNA entropy Distribution with reads <= 3 excluded
Count
0
200
400
600
800
1000
1200
1400
50
100
150
200
250
0.0
0.2
0.4
0.6
0.8
1.0
Normalize Entropy Value
